# An Educational Game Evening for Medical Residents: A Proof of Concept to Evaluate the Impact on Learning of the Use of Games

**DOI:** 10.1128/jmbe.00119-21

**Published:** 2021-07-30

**Authors:** Théo Ghelfenstein-Ferreira, Anne-Lise Beaumont, Sarah Dellière, Nathan Peiffer-Smadja, Nicolas Pineros, Etienne Carbonnelle, Gilbert Greub, Salam Abbara, Liem Binh Luong Nguyen, Mathilde Lescat

**Affiliations:** a Laboratoire de Microbiologie Clinique, Hôpitaux Universitaires de Paris Seine-Saint-Denis Assistance Publique–Hôpitaux de Paris (AP-HP), Bobigny, France; b Service de Maladies Infectieuses, Hôpital Saint Louis, AP-HP, Paris, France; c Laboratoire de Parasitologie-Mycologie, Hôpital Saint-Louis, AP-HP, Paris, France; d Service de Maladies Infectieuses et Tropicales, Hôpital Bichat-Claude-Bernard, AP-HP, Paris, France; e Université de Paris, Paris, France; f INSERM, IAME, Paris, France; g Université Sorbonne Paris Nord, Epinay Villetaneuse, France; h Institut de Microbiologie, Département des Laboratoires, Université de Lausanne, CHUV, Lausanne, Switzerland; i Institut Pasteur, U1018 Inserm, UVSQ, Paris, France; j Centre d’Investigation Clinique, Hôpital Cochin, AP-HP, Paris, France

**Keywords:** educational games, evaluation, feedback

## Abstract

Insufficient knowledge of bacteria and antimicrobials leads to the emergence of multidrug-resistant-bacterium infections. Diversification of the teaching forms, such as the use of games, could be a solution. We organized an event around 3 games (Bacteria Game, KROBS, and Dawaa) to collect student feedback on the evening and assess their knowledge before and after the evening using multiple-choice questions. The preliminary results suggest a positive effect of this event, but due to the low number of participants, we see this report more as a proof of concept to assess the impact of games on the learning.

## INTRODUCTION

The emergence of infections caused by multidrug-resistant bacteria is a major public health problem that is partly due to the misuse or widespread use of antimicrobials (1). Insufficient knowledge of bacteria and of the prescribing of antimicrobials in many categories of professionals is one of the major causes of this misuse (2). Hence, better training is needed to improve antibiotic use and maintain our therapeutic arsenal (3). Most of the teachings in microbiology or antimicrobial therapy are done via formal lectures (4). Educational games appeared to be a particularly interesting tool to diversify teaching methods in medical schools (5). Their instructions and objectives are clear, they pose a challenge for residents, they take place over a short period of time, allow social interactions and collaborations, and are meaningful and interdisciplinary (6). While the idea of using a game to facilitate learning is an old one, there are few studies with rigorous methodology and quantitative measures to assess their teaching effectiveness. One study concerned an infectious disease game that showed a beneficial effect when posttest multiple-choice questions (MCQs) were presented (7).

By organizing educational evenings around microbes and infectious diseases, we aimed to assess the qualitative and quantitative impacts of the first educational “game evening,” using 3 educational games (Bacteria Game, KROBS, and Dawaa), on 16 medical residents (with a specialty in infectious disease or microbiology).

(The results of this study have previously been reported in the meeting of the Webinars SFM 2020 congress of the French Society for Microbiology).

## MATERIALS AND METHODS

### Students and descriptions of games

Sixteen medical residents (in infectious disease and microbiology specialties) participated in an educational game evening co-organized by the Network of Young Clinical Microbiologists (ReJMiC, SFM), the French Network of Young Infectious Diseases Doctors (RéJIF), and the creators of the games. Three educational games targeting medical students and residents were used.

Bacteria Game is a card game created by a microbiologist from the University of Sorbonne (Paris Nord) that aims to consolidate the knowledge of the essential characteristics of 20 bacteria of clinical interest. It can be played alone or under the supervision of a clinical microbiologist. Dawaa is a semicollaborative board game on the use of antibiotics created by infectious disease specialists from the University of Paris. It requires supervision by an infectious disease specialist (8, 9). KROBS is a card game with 20 different microbes, created by a microbiologist from the University of Lausanne (Switzerland). The latter game aims at explaining the major modes of transmission of infectious diseases. This game can be played independently or under the supervision of a microbiologist (10, 11). The students were residents in infectious diseases and microbiology and were distributed in the groups in order to have these two specialties in each group, i.e., four groups of four people (composed of three and one resident, respectively, specializing in infectious diseases and in clinical microbiology). The players rotated among four workshops of 30 min, one per game and one allowing an informal meeting between the residents. During the fourth workshop, participants were free to discuss their experiences of hospital internships in infectious disease departments and microbiology laboratories to allow better communication between future clinical practitioners and microbiologists. The distribution in the groups remained the same all evening.

### Methods of evaluation

The residents completed an educational-evening evaluation consisting of the following 3 questions to which they could give a score of 1 to 7. “Does a serious game evening contribute to your educational training?” “Would educational games be relevant to support diploma training days, through dedicated sessions?” “How would you rate this evening overall?”

An initial evaluation questionnaire (Q-go) and a summative (Q-return) with 7 MCQs of 5 items each were also distributed upon arrival and at the end of the evening, respectively. During these evaluation times, participants could not discuss their answers. The questions were chosen according to the concepts covered in the 3 games used. Similar types of questions were used in both questionnaires to allow comparability of results (see the supplemental material). The scores, expressed as percentages, were obtained by first calculating the sum of correctly checked or not checked answers, from which the sum of wrongly checked or not checked answers had been removed. The scores of the paired Q-go and Q-return questionnaires of the residents were compared using the Wilcoxon test with R software (12). Statistical significance was determined at a *P* value of less than 0.05.

## RESULTS

**FIG 1:**
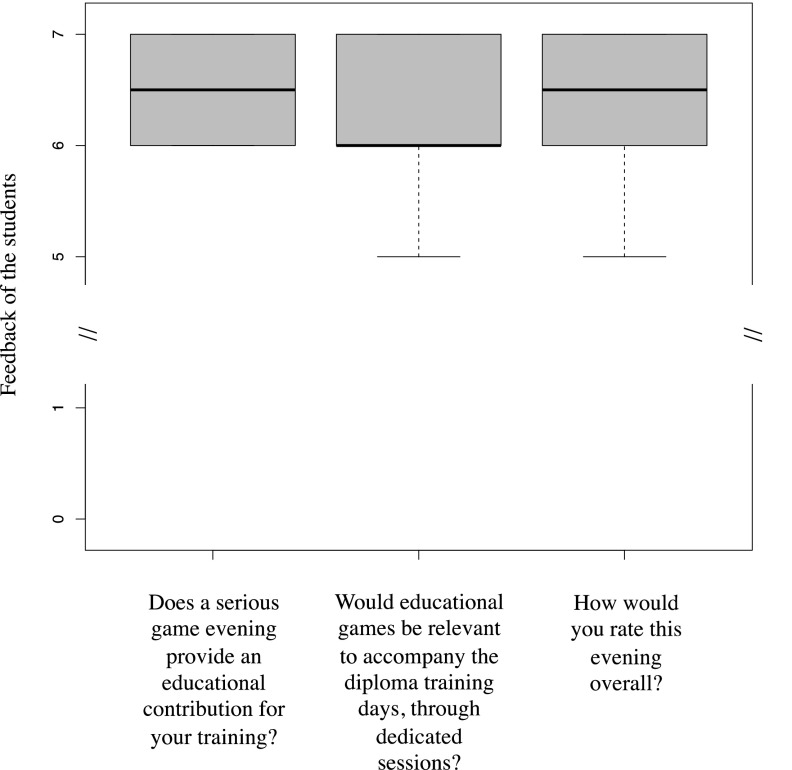
Boxplots of feedback from the residents specializing in infectious diseases and microbiology (*n* = 15) who answered the feedback questionnaire. The feedback questionnaire consisted of the following 3 questions, to which residents could give an evaluation on a score of 1 to 7. “Does a serious game evening contribute to your educational training?” “Would educational games be relevant to accompany the diploma training days, through dedicated sessions?” “How would you rate this evening overall?” Each boxplot corresponds to one question and represents the scores of all residents.

**FIG 2:**
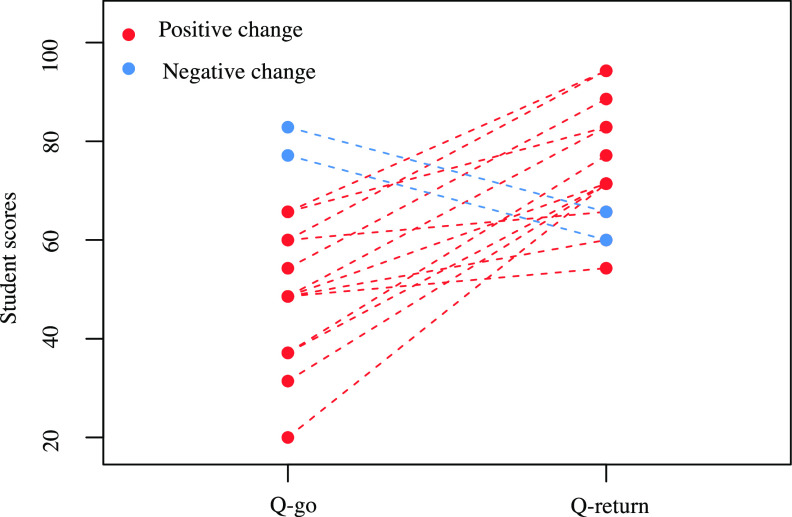
Paired scores for the Q-go and Q-return questionnaires of the participating residents, who specialized in infectious diseases and clinical microbiology (*n* = 15). Positive and negative changes are represented in red and blue, respectively. We considered as True Positive (TP): items checked when they were right; True Negative (TN): items not checked when they were wrong; False Positive (FP): items checked when they were wrong; False Negative (FN): items not checked when they were right. The scores, expressed as percentages, were obtained by the following formula: (((TP + TN) − (FP + FN))/(total number of items)) * 100. The scores of the paired Q-go and Q-return questionnaires of the residents were compared using the Wilcoxon test with R software (12). With this test, we observed higher scores for Q-return than for Q-go questionnaires (*P* = 0.002).

After excluding participants who did not return or did not fully complete their questionnaires (*n* = 1), 15 pairs of Q-go and Q-return could be analyzed. The median age was 25 (range, 24 to 25.75) years. The median level of the residents was 1 (range, 0.5 to 4) year. Four residents were specializing in clinical microbiology, and 11 in infectious diseases. Regarding the evening evaluation questionnaires, all residents rated the event very positively. Favorable and meaningful positive interactions between the participants to the event were observed by the moderators and reported by the participants. All considered that the evening presented a significant educational contribution to their training (50% gave a mark of 6/7, and 50% a mark of 7/7). A large majority (13/15) assessed that educational game sessions could be integrated into their university training course (with a score greater than or equal to 6/7). All participants rated the game evening as “excellent overall” (14 residents out of 15 gave scores higher than or equal to 6/7) (Fig. 1). Overall, there was an increase in scores during the evening regarding questions addressed by the games, with a score of 52% correct answers in the Q-go and 74% in the Q-return (*P* = 0.002) (Fig. 2).

## DISCUSSION AND CONCLUSIONS

The qualitative evaluation of this educational game evening revealed that 15 residents who specialized in microbiology or infectious diseases were interested in such educational events and, more generally, in establishing transdisciplinary collaborations during the games and the breaks. The scores of the residents on an initial and a summative evaluation focusing on microbiology and infectious disease themes were significantly increased, suggesting an impact of the games on the knowledge of the residents. It is clear that the low number of participants does not allow us to formally conclude that the use of these games has an impact on the learning of residents in general. Moreover, we were not able in the single social event to compare the levels of the Q-go and Q-return questionnaires and be sure the increase in the residents’ scores was due to the use of the games. A study with other educational game evenings that would compare the use of games with traditional teaching methods, such as lectures, would make it possible. Such a study would also use two Q-go and Q-return evaluation questionnaires, each containing 10 MCQs drawn from the same bank of questions. In this further study, we would use two series of Q-go and Q-return questionnaires, each with 10 MCQs taken from a question bank. The two series of questionnaires would contain the same Q-go and Q-return MCQs but reversed in order to compare their level. Moreover, this study would allow feedback on each game used in the study. We chose to test these 3 games to allow a mixture of games with very different educational goals. The combination of these 3 games required both clinical and microbiological skills about microbe characteristics, antibiotic therapy, or transmission of microbes. The short duration of the sessions avoided a loss of attention of the participants. Moreover, a 30-minute session was in line with the length of the game for each of these games. Besides, we chose to carry out this social event with residents who already had knowledge of infectious diseases and microbiology in order to consolidate their knowledge. It could be interesting to organize this kind of event with less advanced students, to sensitize a larger and less specialized medical public to the problems of antimicrobial resistance, for instance. In this case, this event could close a module of an infectious diseases class, allowing the students to be able to participate in the games. In conclusion, we see this preliminary work more as a “proof of concept” and we hope that this initiative will help in setting larger studies in the future that will more precisely assess the impact of games on the learning curve of residents.
