# Supplementary material for: An Educational Game Evening for Medical Residents: A Proof of Concept to Evaluate the Impact on Learning of the Use of Games
Source: J Microbiol Biol Educ. 2021 Jul 30;22(2):e00119-21. doi: 10.1128/jmbe.00119-21 (PMC8442010; doi:10.1128/jmbe.00119-21)
Supplement: SUPPLEMENTAL FILE 1 — Download JMBE00119-21_Supp_1_seq2.pdf, PDF file, 0.07 MB [file jmbe00119-21_supp_1_seq2.pdf]

Survey of the educational game event

You are:
☐Woman☐Man

your age: years.

Which year of residency:

Are you specialized
☐ in medical biology in microbiology
☐ Infectious diseases

Have you ever played BACTERIA GAME (if so how many times)?
☐Yes (... times)
☐No

Have you ever played DAWAA (if so how many times)?
☐Yes (... times)
☐No

Have you ever played KROBS (if so how many times)?
☐Yes (... times)
☐No

Q-Go

1 - The *Enterococcus faecium* species (multiple answers possible)
□appears as a chain gram positive cocci
□is part of the Micrococcaceae family
□is usually identified in the laboratory by serology
□usually treated with amoxicillin
□is a commensal of the digestive tract

2 - The species *Escherichia coli* (multiple answers possible)
□has a strict aerobic respiratory character
□can cause diarrhea
□frequently has overproduction of cephalosporinase
□can give meningitis
□is a commensal of the skin

3 – Concerning *Enterobacteriaceae* family(multiple answers possible)
□ Group III E*nterobacteriaceae* are naturally resistant to 3rd generation cephalosporins
□ *Salmonella enterica.* and *Shigella* are part of group 0 *Enterobacteriaceae*
□ Group 1 *Enterobacteriaceae* have a low level, inducible chromosomal cephalosporinase
□ Wild *Enterobacter cloacae* is susceptible to ticarcillin
□ *Klebsiella pneumoniae* is resistant to 1st generation cephalosporin

4 - Among these microbes transmitted by the intake of contaminated food, which one or which are classically associated with the ingestion of raw or undercooked fish flesh (salmon tartare, sushi, etc.)?
□ *Taenia solium*
□ *Taenia saginata*
□ *Diphyllobothrium latum*
□ *Ascaris lumbricoides*
□ *Ancylostoma duodenale*

5 - What is the origin of the name of the *Pasteurella* genus?
□ It is resistant to pasteurization
□ It can contaminate pasteurized milk
□ It is involved in epidemics in sheep and affects shepherds
□ It was named in tribute to Mr. Pasteur
□ It can be found in stagnant water (including holy water and has infected babies at baptism)

6 - Which of these microbes can be transmitted by exposure to a contaminated air conditioning system?
□ *Chlamydia psittaci*
□ *Legionella pneumophila*
□ *Mycobacterium kansasii*
□ *Parachlamydia acanthamoebae*

□ *Lausannevirus*

7 - The species *Neisseria meningitidis* (multiple answers possible)
□ appears as a chain gram negative cocci
□ can be diagnosed by molecular biology in a routine laboratory
□ can be treated with amoxicillin
□ has increased mortality in meningitis
□ gives more invasive infections when encapsulated

Q-Return

1 – Group 3 *Enterobacteriaceae* (multiple correct answers)?
 are Gram-negative coccis
 are upper airway commensals
 are requently have overproduction of cephalosporinase
 are generally responsible for community infections
 are commensals of the digestive tract

2 - *Neisseria gonorrhoeae* (multiple correct answers)
 is a Gram-negative bacillus
 can be diagnosed by culture
 is resistant to β-lactams generally by the production of a penicillinase
 can be diagnosed by molecular biology
 rather gives nosocomial infections

3 *Streptococcus pneumoniae* (multiple answers possible)
 has a particular enzyme, pneumolysin
 is a Gram Positive clumped Cocci
 may look like a Gram negative bacillus if the bacteria was lysed
 is frequently involved in meningitis
 is rarely resistant to amoxicillin

4 - Concerning rifampicin (multiple correct answers)
 the use of rifampicin in monotherapy is not recommended.
 rifampicin diffuses very well into tissues, including bones
 It has anti-staphylococcal activity but not anti-streptococcal
 It has a good diffusion in contact with the material, including in the biofilm
 It is not active on *Staphylococcus aureus*

5 - Among the following modes of transmission, which one or which are involved in certain transmissions of *Coxiella burnetti*, a pathogen of Q fever?
 sleep in a sheepfold.
 getting bitten by a cat
 give birth to a sheep
 eat food contaminated with rat droppings
 being exposed to a parrot

6 - Which or which of these zoonoses are transmitted by aerosols?
 Chlamydia psitacci
 Coxiella burnetii
 Lassa virus
 Rhabdochlamydia helvetica
 Mycobacterium kansasii

7 - Which of the pathogens below belong to the Spirochetes family?
 Treponema pallidum
 Bartonella henselae
 Leptospira interogans
 Borrelia burgdorferi
 Pasteurella multocida
